# Estimating age‐dependent survival when juveniles resemble females: Invasive ring‐necked parakeets as an example

**DOI:** 10.1002/ece3.4366

**Published:** 2019-02-05

**Authors:** Juan Carlos Senar, Lluïsa Arroyo, Alba Ortega‐Segalerva, José G. Carrillo, Xavier Tomás, Tomas Montalvo, Ana Sanz‐Aguilar

**Affiliations:** ^1^ Natural History Museum of Barcelona Barcelona Spain; ^2^ Agencia de Salut Pública de Barcelona Barcelona Spain; ^3^ CIBER de Epidemiología y Salud Pública Barcelona Spain; ^4^ Animal Demography and Ecology Group IMEDEA, CSIC‐UIB Esporles Spain

**Keywords:** age‐specific survival, capture–recapture, delayed plumage maturation, multievent models, ring‐necked parakeet, survival

## Abstract

Many species only show sexual dimorphism at the age of maturity, such that juveniles typically resemble females. Under these circumstances, estimating accurate age‐specific demographic parameters is challenging. Here, we propose a multievent model parameterization able to estimate age‐dependent survival using capture–recapture data with uncertainty in age and sex assignment of individuals. We illustrate this modeling approach with capture–recapture data from the ring‐necked parakeet *Psittacula krameri*. We analyzed capture, recapture, and resighting data (439 recaptures/resightings) of 156 ring‐necked parakeets tagged with neck collars in Barcelona city from 2003 to 2016 to estimate the juvenile and adult survival rate. Our models successfully estimated the survival probabilities of the different age classes considered. Survival probability was similar between adults (0.83, 95% CI = 0.77–0.87) and juveniles during their second (0.79, 95% CI = 0.58–0.87) and third winter (0.83, 95% CI = 0.65–0.88). The youngest juveniles (1st winter) showed a slightly lower survival (0.57, 95% CI = 0.37–0.79). Among adults, females showed a slightly higher survival than males (0.87, 95% CI = 0.78–0.93; and 0.80, 95% CI = 0.73–0.86, respectively). These high survival figures predict high population persistence in this species and urge management policies. The analysis also stresses the usefulness of multievent models to estimate juvenile survival when age cannot be fully ascertained.

## 1 INTRODUCTION

Many life history processes and parameters are age‐dependent (e.g., age at maturity, age‐specific survival, or age‐specific reproductive investment) (Roff, 1992; Stearns, 1992). Accordingly, the age of an individual is a key ecological parameter in population dynamics studies (Cam, 2009; Perrins, Lebreton, & Hirons, 1991; Sutherland, 1996; Williams, Nichols, & Conroy, 2002). However, too often, the age of the individuals cannot be easily ascertained. The topic is further complicated in species in which juveniles resemble females, such that uncertainty appears not only in relation to the age of a high proportion of the individuals, but also in relation to their sex (Busse, 1984; Jenni & Winkler, 1994; Pyle, Howell, Yunick, & DeSante, 1987; Svensson, 1992). A general approach for studying age‐ and sex‐specific population dynamics parameters in these species is to consider only the individuals for which age and sex have been determined with reasonable certainty. However, this approach may entail discarding substantial data. Even more importantly, this approach can bias data; since by definition, we use only individuals that have survived long enough to allow the determination of their sex/age.

Multievent capture–recapture models (Pradel, 2005) have been successfully used to estimate demographic parameters when there is uncertainty in the individuals’ assignment to a particular sex (Genovart, Pradel, & Oro, 2012), breeding status (Desprez, McMahon, Hindell, Harcourt, & Gimenez, 2013), health state (Conn & Cooch, 2009), or behavioral characteristics (Sanz‐Aguilar, Jovani, Melián, Pradel, & Tella, 2015). Here, we propose a multievent model parameterization able to estimate age‐dependent survival using capture–recapture data with uncertainty in age and sex assignment of individuals. We illustrate this modeling approach with capture–recapture data from the ring‐necked parakeet *Psittacula krameri*. Male ring‐necked parakeets over 3 years old can easily be sexed due to males’ rose‐colored neck‐rings and black bibs (Butler & Gosler, 2004). However, although the shape of the primaries and the amount of yellow in undercovers has been suggested to discriminate adults and juveniles (Butler & Gosler, 2004), the differences are far from clear. Thus, immature males and both immature and adult females are, in practice, highly monomorphic. The species thus exemplifies a typical case of uncertainty in the age and sex of a proportion of the population.

The ring‐necked parakeet is also interesting as it is an invasive, exotic, pest bird species that has established feral populations in many temperate regions in Europe, North America, and Asia (Jackson et al., 2015; Le Gros et al., 2016; Strubbe, Jackson, Groombridge, & Matthysen, 2015; Strubbe & Matthysen, 2009). It is considered a pest in most of these newly established areas as it is known to cause agricultural damage and noise pollution and to compete with some native species (Covas, Senar, Roqué, & Quesada, 2017; Hernández‐Brito, Carrete, Popa‐Lisseanu, Ibáñez, & Tella, 2014; Menchetti & Mori, 2014). Recent studies have estimated ring‐necked parakeet breeding success (Braun, 2004; Butler, Cresswell, Gosler, & Perrins, 2013) and dispersal (Braun, 2009). However, the age‐dependent survival probabilities of the species, which are key parameters to estimate the intrinsic rate of increases in its populations (Butler et al., 2013), are still unknown.

The aim of this study is twofold: (a) to provide a multievent model approach to overcome the problem, typical to many species, of uncertainty in age and sex determination of individuals when estimating age‐dependent survival probabilities; and (b) to provide estimates of age‐dependent survival rates (and sex‐dependent in the case of adults) for the ring‐necked parakeet, which can be of use in population dynamics and viability models, essential to evaluating the risks of invasion of this species (Pruett‐Jones, Newman, Newman, Avery, & Lindsay, 2007).

## 2 MATERIALS AND METHODS

The study was conducted in the city of Barcelona, Spain. The ring‐necked parakeet became established in Barcelona in the early 1970s (Batllori & Nos, 1985), and the population has steadily increased to the current estimate of about 800 individuals (Senar, Montalvo, Pascual, & Arroyo, 2017). Capture and recapture of ring‐necked parakeets were conducted using a modified Yunick Platform Trap (2 × 1 × 1 m; (Yunick, 1971)) located at the Natural History Museum of Barcelona. The Museum is located in Ciutadella Park, which hosts one of the largest ring‐necked parakeet colonies in the city (Senar et al., 2017). Between spring 2003 and spring 2016, we tagged a total of 156 individuals with metal rings as well as aluminum neck collars with numbered tags that could be read without having to trap the bird (Senar, Carrillo‐Ortiz, & Arroyo, 2012). During the study period, we obtained 157 recaptures and collected 282 resightings of the numbered birds from several sources: via transects conducted in Ciutadella Park to locate the birds, via reports from birdwatchers in Barcelona, and via observations made during the course of other activities, such as censuring Monk parakeets (*Myiopsitta monachus*). The resightings were pooled with recaptures to obtain a better estimate of parameters. Parakeets were captured, recaptured, or resighted on average 3.9 times (*SD*: 4.1), with great variation between individuals (a range of 0–26 reobservations per individual). As the estimation of annual survival rates requires short sampling periods, only birds recorded between December and April each year were used. This is also the period in which more parakeets are trapped. The sample sizes and values provided refer to that period.

We only distinguished two classes of birds in the field: uncertain plumaged birds (coded “1”; which could be immature males, immature females, or adult females) and adult males (coded “2”; over 3 years old). However, capture–encounter histories *per se* contain additional information that informs the model about individual's age and sex (i.e., the real biological state). For example, an encounter history “10122220000000” belongs to a male marked as 1st winter juvenile. On the contrary, an encounter history “00000101100101” belongs to a female captured and marked with uncertain age, but clearly adult after its 2nd resighting.

Survival probabilities were modeled by means of multievent capture–recapture models accounting for uncertainty in young and adult female identification. The multievent framework distinguishes what can be observed in the field (the events coded in the encounter histories) from the underlying true biological states of the individuals, which must be inferred (Pradel, 2005). Our model included six biological states: 1st winter juvenile parakeet alive (coded J1), 2nd winter juvenile parakeet alive (coded J2), 3rd winter juvenile parakeet alive (coded J3), adult female parakeet alive (coded F), adult male parakeet alive (coded M), and parakeet locally dead (coded D). Encounter histories were coded using three different events (see below). Each row of encounter histories belonged to a different individual, and each column referred to year. The three events used were as follows:

Event “0” was used to indicate that the individual was not captured/resighted at a particular time point.

Event “1” was used to indicate that the individual was captured/resighted at a particular time point and showed a female/young plumage.

Event “2” was used to indicate that the individual was captured/resighted at a particular time point and showed an adult male plumage (i.e., evident neck collar).

Multievent models use three kinds of parameters: the initial state probabilities, the transition probabilities, and the event probabilities (conditional on the underlying states).

The initial state probabilities correspond in our model to the proportions of newly tagged individuals belonging to the different states (i.e., proportions of 1st, 2nd, and 3rd year juveniles, adult females, and adult males at first capture, vector (Vector 1)). We did not consider temporal variation in initial state probabilities.

(Vector 1) $$\begin{array}{cc} & \;\begin{array}{ccccccccc} \text{J1} & & \text{J2} & & \;\;\text{J3} & & \;\;F & & \;\;\;M \end{array}\\ \text{Initial}\_\;\text{State} & =\left(\begin{array}{ccccccccc} \pi_{1j} & & \pi_{2j} & & \pi_{3j} & & \pi_{f} & & 1-\pi_{1j}-\pi_{2j}-\pi_{3j}-\pi_{f} \end{array}\right) \end{array}$$

The transition probabilities correspond to survival and transition between state processes and were modeled in two steps. The first step accounted for survival *Φ* and mortality 1 ‐ *Φ* probabilities (matrix (Matrix 1)). Here, we tested the effects of time, age and sex (only for adults) on survival using different parameter structures, as follows: We allowed for differences between 1st winter birds and older birds together, so that we assumed that once the birds have reached their second year, they enjoy a survival rate similar to adult birds (J1, J2 = J3 = AD); we considered the same survival for 1st and 2nd winter birds (i.e., young juveniles) but different from the others together with 3rd year birds and adults being equal (J1 = J2, J3 = AD); we allowed for differences between 1st winter, 2nd and 3rd winter (being equal), and adults (J1, J2 = J3, AD); we considered differences between juveniles (all ages together) and adults (J1 = J2 = J3, AD); and we considered a constant model with no differences between juveniles and adults in survival rate, so that survival is independent of age (.). Finally, we additionally tested the effect of sex on adult survival using two possible age structures for juveniles: (J1, J2 = J3, F, M) and (J1 = J2 = J3, F, M). Note that survival and mortality probabilities estimated in this way must be considered local/apparent (i.e., they do not allow for distinguishing between mortality and permanent emigration).

(Matrix 1) $$\begin{array}{cc} & \;\;\begin{array}{ccccccccccc} \text{J1} & & \text{J2} & & \;\;\text{J3} & & \;\;\;\;F & & \;M & & \;D \end{array}\\ \text{Survival} & =\begin{array}{c} \text{J1}\\ \text{J2}\\ \text{J3}\\ F\\ M\\ D \end{array}\left(\begin{array}{ccccccccccc} \phi_{1j} & & 0 & & 0 & & 0 & & 0 & & 1-\phi_{1j}\\ 0 & & \phi_{2j} & & 0 & & 0 & & 0 & & 1-\phi_{2j}\\ 0 & & 0 & & \phi_{3j} & & 0 & & 0 & & 1-\phi_{3j}\\ 0 & & 0 & & 0 & & \phi_{\mathrm{ad}} & & 0 & & 1-\phi_{\mathrm{ad}}\\ 0 & & 0 & & 0 & & 0 & & \phi_{\mathrm{ad}} & & 1-\phi_{\mathrm{ad}}\\ 0 & & 0 & & 0 & & 0 & & 0 & & 1 \end{array}\right) \end{array}$$

In a second step and conditional on individual survival, we modeled transition between states *ψ* probabilities (matrix (Matrix 2)). As the period between capture/resight occasions lasted 1 year, surviving juveniles of 1st and 2nd winter move to the next age class (matrix (Matrix 2)) and juveniles of 3rd winter become adults (males or females) with the same probability (i.e., assuming that sex ratio is balanced in the population, *ψ* = 0.5). This assumption is critical to making the model parameters identifiable.

(Matrix 2) $$\begin{array}{cc} & \;\begin{array}{ccccccccccc} \;\text{J1} & & \text{J2} & & \text{J3} & & F & & M & & \;\;\;D \end{array}\\ \text{Transition} & =\begin{array}{c} \text{J1}\\ \text{J2}\\ \text{J3}\\ F\\ M\\ D \end{array}\left(\begin{array}{ccccccccccc} 0 & & 1 & & 0 & & 0 & & 0 & & 0\\ 0 & & 0 & & 1 & & 0 & & 0 & & 0\\ 0 & & 0 & & 0 & & \psi & & 1-\psi & & 0\\ 0 & & 0 & & 0 & & 1 & & 0 & & 0\\ 0 & & 0 & & 0 & & 0 & & 1 & & 0\\ 0 & & 0 & & 0 & & 0 & & 0 & & 1 \end{array}\right) \end{array}$$

The event probabilities relate the observations coded in the capture histories (columns) to the underlying biological states (rows). Here, we modeled the probability of resighting (p, matrix (Matrix 3)). We did not consider age or sex effects on resighting probabilities because as we used resightings in addition to recaptures, we did not have a priori reasons to expect age or sex effects of resighting probabilities. Additionally, we had insufficient data to estimate a more complex model, which is apparent from the fact that trying these models produced a CI in parameter estimators that were too large, reducing confidence in these models.

(Matrix 3) $$\begin{array}{cc} & \;\;\begin{array}{ccccc} 0 & & \;1 & & 2 \end{array}\\ \text{Resighting} & =\begin{array}{c} \text{J1}\\ \text{J2}\\ \text{J3}\\ F\\ M\\ D \end{array}\left(\begin{array}{ccccc} 1-p & & p & & 0\\ 1-p & & p & & 0\\ 1-p & & p & & 0\\ 1-p & & p & & 0\\ 1-p & & 0 & & p\\ 1 & & 0 & & 0 \end{array}\right) \end{array}$$

The overall goodness‐of‐fit test of the Jolly Movement model for multistate data was calculated using U‐CARE2.3.2 (Choquet, Lebreton, Gimenez, Reboulet, & Pradel, 2009; Choquet, Rouan, & Pradel, 2009) and was not statistically significant (Table 1). Overdispersion was not apparent (χ^2 ^= 45.82, 60 *df*,* p* = 0.91), and thus, there was no indication of violation of the assumption that fates of the individuals were independent of each other (Anderson, Burnham, & White, 1994).

**Table 1:** Results of the goodness‐of‐fit test of the Jolly Movement model for multistate data calculated using U‐CARE2.3.2. Data showed a good fit to a general CR model

TEST	χ^2^	*df*	*p*‐value
3G.SR	16.910	18	0.53
3G.SM	9.640	24	0.99
M.ITEC	13.398	11	0.27
M.LTEC	5.873	7	0.56
Total	45.821	60	0.91

Parameters were estimated simultaneously by maximum likelihood using the program E‐SURGE 1.6.3 (Choquet, Rouan & Pradel, 2009). Model selection was based on Akaike's information criterion adjusted for the effective sample size (AICc) (Anderson et al., 1994; Burnham & Anderson, 2002). Models with AICc values differing by less than 2 were considered equivalent. We only tested additive temporal effects on survival to avoid overparameterized models. Estimates were obtained by model averaging using Akaike weights (Burnham & Anderson, 2002).

Mean life span was estimated from survival rate according to Mean life span = (1/(‐LN(Survival rate))) (Brownie, Anderson, Burnham, & Robson, 1985).

## 3 RESULTS

**Figure 1:**
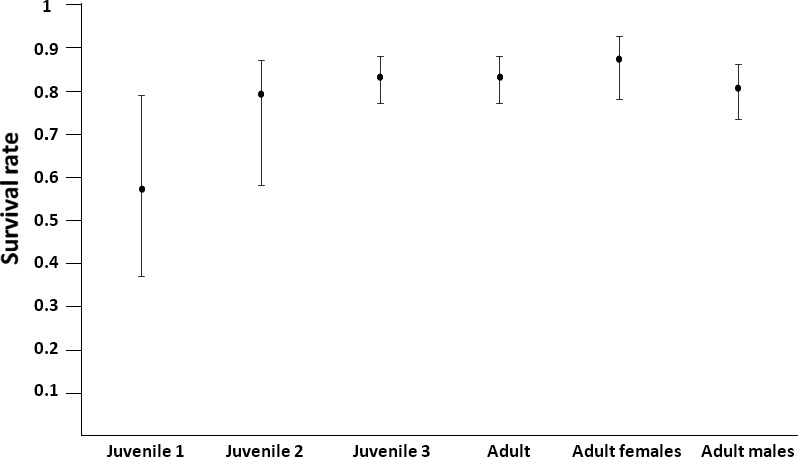
Model‐averaged estimates of juvenile (aged 1 to 3; Models 1–10, Table 2), adult (both sexes; Models 1–10, Table 2), adult females, and adult males (Table 3) survival probabilities of ring‐necked parakeets at Barcelona (2003–2016)

Models with time‐dependent variation in resight probability were not better than models with constant resight probability (Tables 2 and 3). The model with the lowest AICc assumed a constant resight probability (0.44, 95% CI = 0.38–0.50, Model 21, Table 3). Survival probability was modeled according to different age structures, differing in how we pooled ages (Table 2) and with and without sex effects for adult birds (Table 3). Models with temporal variation of survival were not retained (Table 2). In general, models with different age structures were close in terms of AICc (Table 2) indicating no significant differences in survival probabilities among the different age classes (Table 2, Figure 1). Regarding age‐dependent survival, model‐averaged survival estimates (Models 1 to 10, Table 2; Figure 1) were as follows: 0.57 (95% CI = 0.37–0.79) for first year juveniles, 0.79 (95% CI = 0.58–0.87) for second year juveniles, 0.83 (95% CI = 0.77–0.88) for third year juveniles and 0.83 (95% CI = 0.77–0.87) for adults (Models 1–10, Table 2). Regarding sex effects on adult survival, models with sex effects were preferred but they were very close in terms of AICc to models without sex effects (Table 3). Model‐averaged survival estimates (Table 3) were as follows: 0.87 (95% CI = 0.78–0.93) for adult females and 0.80 (95% CI = 0.73–0.86) for adult males. Overall survival rate for the species was estimated at 0.81 (95% CI = 0.77–0.85) (Model 5, Table 2), and mean life span was estimated at 4.8 years (95% CI = 3.6–6.4 years). Longevity records were for one individual that reached 14 years of life and another one that reached 12 years.

**Table 2:** Model selection for age and time effects on survival probability and time effects of recapture probability (*p*) of ring‐necked parakeets in Barcelona (2003–2016)

Model	Survival	*p*	np	Deviance	AICc	ΔAICc
1	J1, J2 = J3 = AD	.	7	1,046.19	1,060.53	0
2	J1, J2 = J3 = AD	Time	19	1,020.37	1,060.78	0.25
3	J1 = J2, J3 = AD	.	7	1,047.13	1,061.47	0.94
4	J1 = J2, J3 = AD	Time	19	1,021.25	1,061.65	1.13
5	.	.	6	1,049.46	1,061.72	1.19
6	J1, J2 = J3, AD	.	8	1,045.52	1,061.96	1.43
7	J1 = J2 = J3, AD	.	7	1,047.87	1,062.21	1.68
8	.	Time	18	1,024.17	1,062.33	1.80
9	J1 = J2 = J3, AD	Time	19	1,022.08	1,062.49	1.96
10	J1, J2 = J3, AD	Time	20	1,019.92	1,062.59	2.06
11	J1, J2 = J3 = AD + Time	.	19	1,023.28	1,063.69	3.16
12	J1, J2 = J3, AD + Time	Time	20	1,021.26	1,063.92	3.40
13	J1, J2 = J3 = AD + Time	Time	31	997.92	1,066.44	5.91
14	J1 = J2, J3 = AD + Time	Time	31	998.83	1,067.36	6.83
15	J1, J2 = J3, AD + Time	Time	32	997.87	1,068.84	8.31
16	J1 = J2, J3 = AD + Time	.	19	1,030.54	1,070.94	10.41
17	J1 = J2 = J3, AD + Time	.	19	1,031.11	1,071.52	10.99
18	Time	.	18	1,033.41	1,071.57	11.04
19	J1 = J2 = J3, AD + Time	Time	31	1,007.31	1,075.83	15.31
20	Time	Time	28	1,016.16	1,077.45	16.92

Note. Models are ranked according to ΔAICc values. Notation: Time: parameters are allowed to change between capture occasions; (.): Parameters independent of time; J1, J2, and J3 refer to juveniles in their 1st, 2nd, or 3rd year, and AD refers to adult birds, from their 3rd year on.

**Table 3:** Model selection for sex effects on survival probability of adult ring‐necked parakeets in Barcelona (2003–2016)

Model	Survival	*p*	np	Deviance	AICc	ΔAICc
21	J1 = J2 = J3, F, M	.	8	1,043.92	1,060.36	0.00
22	J1 = J2 = J3, F, M	Time	20	1,018.09	1,060.76	0.39
23	J1, J2 = J3, F, M	.	9	1,042.73	1,061.29	0.92
6	J1, J2 = J3, AD	.	8	1,045.52	1,061.96	1.60
24	J1, J2 = J3, F, M	Time	21	1,017.05	1,062.00	1.63
7	J1 = J2 = J3, AD	.	7	1,047.87	1,062.21	1.85
9	J1 = J2 = J3, AD	Time	19	1,022.08	1,062.49	2.13
10	J1, J2 = J3, AD	Time	20	1,019.92	1,062.59	2.23

Note. Models are ranked according to ΔAICc values. Notation: Time: parameters are allowed to change between capture occasions; (.): Parameters independent of time; J1, J2, and J3 refer to juveniles in their 1st, 2nd, or 3rd year; AD refers to adult birds of both sexes, from their 3rd year on; F refers to adult females, and M refers to adult males.

## 4 DISCUSSION

Multievent models were developed to specifically account for uncertainty in state assessment in capture–recapture studies (Pradel, 2005). The method has been used since then to estimate demographic parameters with uncertainty in assignment of many different states such as sex, breeding status, or health status (see Pradel (2009) for a review). In this study, we show, for the first time, that the method can be extended to estimate age‐specific survival rates when age cannot be fully ascertained. This is typically the case of species only showing sexual dimorphism at the age of maturity, where juveniles typically resemble females, displaying what has been defined as “delayed plumage maturation” (Butcher & Rohwer, 1989; Senar, 2006). Our approach allows the use of all available data to estimate age‐specific survival probabilities. However, our approach relies on a critical assumption: The sex ratio must be fixed in the model to make parameters identifiable. Moreover, parameters in our multievent model are identifiable because: 1) Age in males can be assessed with certitude when they are marked as juveniles and later recaptured or resighted as adults, and 2) individuals must change their state (age) on an annual basis with all the males acquiring their adult plumage at the same age. Our results suggest that ring‐necked parakeets may suffer higher mortality probability during their first year than older birds. This is a common phenomenon in most bird species (Newton, 1998), including parakeets (e.g., Monk parakeet: 1st year 0.61, adults 0.81 (Bucher, Martin, Martella, & Navarro, 1991); Puerto Rican parrot: 1st year 0.68, adults 0.85 (Snyder, Wiley, & Kepler, 1987)). Alternatively, juveniles during their first year may permanently disperse from the study area in higher proportions than other juveniles and adult birds; unfortunately, very little is known about dispersal patterns in this species.

The fact that models with different age structures were close in terms of AICc may indicate that in our population there are no substantial differences in survival probabilities between age classes. This could be due to the fact that we used birds captured in winter (December–April). If the higher mortality filter occurs soon after fledging and thus before our first captures, our data would be unable to clearly detect this mortality (Payo‐Payo, Genovart, Bertolero, Pradel, & Oro, 2016). Alternatively, in the city, early mortality could be less marked than in the wild (Rebolo‐Ifrán et al., 2015), but again, this hypothesis should be further tested. On the other hand, although our modeling approach allows the estimation of juvenile survival, our estimates are based on uncertain data and consequently are expected to be more uncertain than estimates obtained using encounter data of known‐age individuals.

In general, males enjoy a higher survival rate than females, either because of being subordinate to males or because of a higher parental effort than males (András & Tamás, 2007; Donald, 2007; Promislow, Montgomerie, & Martin, 1992). However, this was not the case in ring‐necked parakeets, in which we found that females enjoy a slightly higher survival rate than males. This could be the result of intense competition between males (András & Tamás, 2007; Promislow et al., 1992) or even, given the apparent stable pair bond of the pair, from protection of females on the part of males (Senar & Domenech, 2011).

Estimating survival probabilities can be critical in alien invasive species to model their expansion rate (Conroy & Senar, 2009; Neubert & Caswell, 2000). Using the multievent model approach, the overall adult survival probability of ring‐necked parakeets in the city of Barcelona was estimated at 0.81. This value is very similar to the survival probability of about 0.80 previously found for the monk parakeet in the same area (Conroy & Senar, 2009). It could be argued that survival of birds in cities is higher than in natural habitats because of the lower predation rate in these urban habitats (Chamberlain et al., 2009; Fischer, Cleeton, Lyons, & Miller, 2012; Møller, 2009; Rebolo‐Ifrán et al., 2015; Walter, Fischer, Baruch‐Mordo, & VerCauteren, 2011). In Barcelona city, some instances of predation by peregrine falcons *Falco peregrinus* and yellow‐legged gulls *Larus cachinnans* on both parakeets have been recorded (J.Quesada *pers.comm*., E.Durany *pers.comm.,* and *pers.obs*.) but these are considered mostly anecdotal compared with the predation rates the birds can suffer in the wild. Accordingly, the survival rate of some other similar‐sized psittacids in the wild show lower survival rates than ring‐necked and monk parakeets (*Amazona finschi* 0.73, *Forpus passerinus* 0.57; reviewed in Senar et al. (2012)). However, data from other similar wild species are also within the range found in the urban populations (*Amazona vittata* 0.89, *Cacatua pastinator* 0.93–0.94, *Cacatua leadbeateri* 0.81–0.93; reviewed in Senar et al. (2012)), including data on wild monk parakeets (0.81, Bucher et al. (1991)). This suggests that the high survival probabilities estimated here for ring‐necked parakeets may be an inherent characteristic of the species rather than a simple consequence of urban life. Unfortunately, data from wild populations are lacking and we cannot distinguish between the two hypotheses.

In summary, our multievent model approach has been shown to be successful to estimate age‐specific survival probabilities for species in which juveniles resemble females, such that uncertainty appears not only in relation to the age of a high proportion of the individuals, but also in relation to their sex. The method allows us to incorporate what could otherwise be defined as “imperfect data” (Desprez et al., 2013) into demographic analyses. In our example, we have been able to provide the first age‐dependent survival estimates for the ring‐necked parakeet, allowing us to predict a high increase in invasive populations of this species (Butler et al., 2013). We therefore strongly advocate for the use of this multievent approach in the estimation of survival rate in species with delayed plumage maturation. Moreover, the method could be adapted to other situations allowing the incorporation of additional information (e.g., data on birds through molecular sexing, intermediate plumages, reproductive behaviors, or the presence of brood patches) even if an exact age or sex determination cannot be made (Genovart et al., 2012). This type of additional information could reduce the uncertainty in the state assignment, and the precision of the estimates can be improved.

## CONFLICT OF INTEREST

None declared.

## AUTHOR CONTRIBUTIONS

The study was conceived by JCS, LA, TM, and ASA. The data were collected by JCS, LA, AOS, JGC, XT, and TM. The statistical analyses were conducted by JCS and ASA. The manuscript was written by JCS, LA, and ASA, with input from all other coauthors. JCS and TM contributed to funding and materials.

## Data Availability

All data used in this study are available in Dryad.
